# Hot electrons in a nanowire hard X-ray detector

**DOI:** 10.1038/s41467-020-18384-x

**Published:** 2020-09-18

**Authors:** Maximilian Zapf, Maurizio Ritzer, Lisa Liborius, Andreas Johannes, Martin Hafermann, Sven Schönherr, Jaime Segura-Ruiz, Gema Martínez-Criado, Werner Prost, Carsten Ronning

**Affiliations:** 1 Institute of Solid State Physics, Friedrich Schiller University of Jena, Max-Wien-Platz 1, 07743 Jena, Germany; 2 Department Components for High Frequency Electronics and CENIDE, University of Duisburg‐Essen, Lotharstr. 53, 47057 Duisburg, Germany; 3 ESRF—The European Synchrotron, 71 Avenue des Martyrs, Grenoble, 30843 France; 4 Instituto de Ciencia de Materiales de Madrid, Consejo Superior de Investigaciones Científicas, Sor Juana Inés de la Cruz 3, 28049 Cantoblanco, Spain

**Keywords:** Electronic devices, Nanosensors, Electronic properties and materials, Nanowires, X-rays

## Abstract

Nanowire chip-based electrical and optical devices such as biochemical sensors, physical detectors, or light emitters combine outstanding functionality with a small footprint, reducing expensive material and energy consumption. The core functionality of many nanowire-based devices is embedded in their p-n junctions. To fully unleash their potential, such nanowire-based devices require – besides a high performance – stability and reliability. Here, we report on an axial p-n junction GaAs nanowire X-ray detector that enables ultra-high spatial resolution (~200 nm) compared to micron scale conventional ones. In-operando X-ray analytical techniques based on a focused synchrotron X-ray nanobeam allow probing the internal electrical field and observing hot electron effects at the nanoscale. Finally, we study device stability and find a selective hot electron induced oxidization in the n-doped segment of the p-n junction. Our findings demonstrate capabilities and limitations of p-n junction nanowires, providing insight for further improvement and eventual integration into on-chip devices.

## Abstract (web summary)

Designing efficient nanowire chip-based electrical and optical devices remains a challenge. Here, the authors present an axial p-n junction GaAs nanowire X-ray detector that enables achieving a spatial resolution of 200 nm; probing the internal electrical field and observing hot electron effects at the nanoscale.

## Introduction

The fundamental optical, electrical, and mechanical properties of semiconductor nanowires were studied in great detail within the past two decades^1–5^. These efforts were motivated by the envisaged use of semiconductor nanowires as building blocks for nanophotonic and nanoelectronic devices^6–9^. Many nanowire-based devices, such as light emitting diodes (LEDs), solar cells, and detectors, are based on p–n junctions^10–13^. Recently, low-cost and high-throughput Aerotaxy production of axial GaAs nanowire p–n junctions was established, proving enormous potential for the industrial production of such nanowire devices^14^.

Technological applications, however, require—beyond a cost-efficient production and a deep understanding of the underlying properties—device stability and reliability. Therefore, key concepts like the nanowire device stability and its interaction with changing environmental conditions need to be realistically considered for the final integration into nanophotonic or nanoelectronic systems^4,15,16^. So far, nanoscale devices have proven to be challenging experimental targets in terms of long-time stability and reliability^4,17,18^. Strong device-to-device variations, huge surface-to-volume ratios, and reduced thermal conductivity make nanowires extremely sensitive to environmental changes necessitating a sophisticated heat management^19,20^.

Here, we report on a single GaAs nanowire axial p–n junction working as a hard X-ray detector with ultra-high spatial resolution. This system is thoroughly analyzed in-operando via nanoscale X-ray analytical techniques. Applying bias voltages in reverse direction allows increasing the efficiency but ultimately leads to device alteration, which yields the limit of the device performance. By combining in-operando X-ray fluorescence (XRF), X-ray absorption near-edge spectroscopy (XANES), and X-ray beam induced current (XBIC) measurements using a synchrotron X-ray nanobeam, we obtain new insights into the hot electron processes involved at the nanoscale.

## Results

### X-ray detection

**Fig. 1:**
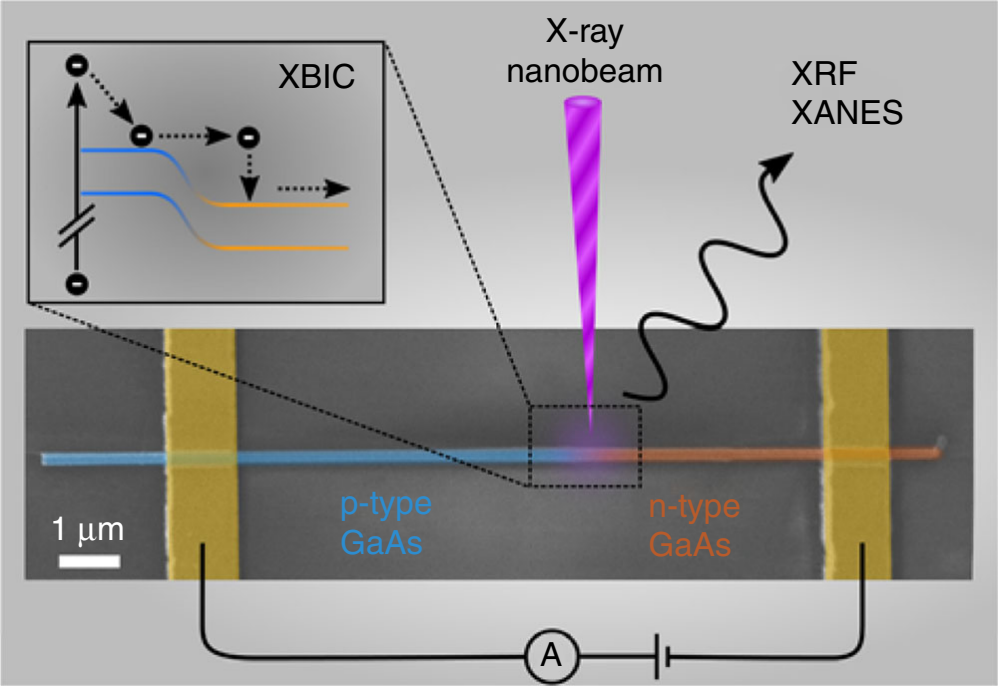
Scheme of the nanowire X-ray detector device. Colorized scanning electron microscope image schematically depicting the device with the experimental setup. An energy-tunable hard X-ray beam was focused onto the GaAs nanowire with a p–n junction along the growth axis and a diameter of 150 nm. The nanowire was contacted, which allowed for in-operando X-ray beam induced current (XBIC, see inset), X-ray fluorescence (XRF), and X-ray absorption near-edge spectroscopy (XANES) measurements while applying external voltages.

Our nanowire device consisted of an axial GaAs nanowire p–n homojunction (shown in Fig. 1). The p and n-type sections were doped with Zn and Sn during gold-catalyzed vapor–liquid–solid growth in a metal organic vapor phase epitaxy (MOVPE) reactor resulting into doping concentrations of ~6 × 10^18^ cm^−3^, respectively. The resulting axial p–n junction nanowire was transferred to a SiO_2_/Si substrate and contacted via two e-beam lithography and metal evaporation steps (details in Supplementary Note 1). This configuration allowed to simultaneously measure XRF and XBIC while applying an external bias voltage. The device was raster scanned across a hard X-ray nanobeam focused to a spot size of 80 × 90 nm^2^ at the nano-analysis beamline ID16B of the European Synchrotron Radiation Facility (ESRF) located in Grenoble, France^21^. In-operando XRF spectra were acquired at different incident X-ray beam energies around the Ga K-absorption edge, while recording the XBIC signal simultaneously. All experiments were conducted under ambient conditions. The current–voltage characteristics were repeatedly measured to monitor the device operation during the measurements (see Supplementary Fig. 3).

**Fig. 2:**
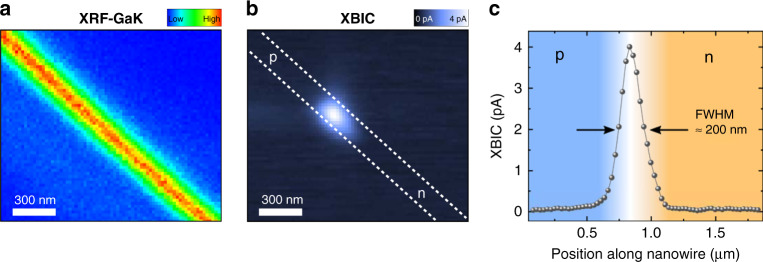
Simultaneous detection of nano XRF and nano XBIC. **a** X-ray fluorescence (XRF) map of a GaAs nanowire depicting the Ga K_α_ line intensity measured with an incident X-ray energy of 11.9 keV, i.e., above the Ga and As K-edges. **b** The corresponding X-ray beam induced current (XBIC) signal at 0 V bias voltage measured simultaneously. The GaAs nanowire (as located in the Ga map) is indicated by the dashed, white lines. **c** Line scan along the nanowire axis of the XBIC signal.

Scanning, high resolution in-operando XRF/XBIC measurements were taken in a selected region around the p–n junction. The nanowire was easily located by the Ga XRF signal (Fig. 2a), while the p–n junction appears bright in the XBIC map (Fig. 2b). The XBIC signal results from the electron–hole pairs created in the de-excitation cascade of hot electrons excited above the conduction band by the impinging X-rays^22^. If electron–hole pairs are generated in the depletion zone of the p–n junction, the internal electrical field efficiently separates the electron–hole pair and drifts the electrons through the junction toward the n-side and the contacts, where they are measured as a current signal. This enables detecting hard X-rays with extremely high spatial resolution at zero bias. The length of the depletion zone of the p–n junction and the nanowire diameter determine the spatial resolution. The measured XBIC signal is finally determined by the convolution of the detector resolution and the spot size of the focused hard X-ray nanobeam. Nanowire-based hard X-ray detectors were already reported with outstanding performance in terms of spatial resolution and XBIC efficiency compared to their bulk counterparts^23–25^. In particular, a spatial resolution of 0.51 µm was achieved for InP nanowires^23^, much higher than in conventional detectors with pixel sizes of tens of microns^26^. Yet, in order to reach such a resolution, sophisticated and computationally demanding ptychographic reconstruction techniques^27^ were required.

Remarkably, our GaAs nanowire X-ray detector shows a direct imaging FWHM of the XBIC signal along the nanowire of only ~200 nm (Fig. 2c). Since the spatial extent of the focused X-ray nanobeam is considerably smaller, it does not contribute much to the width of the measured signal. We include it in the detector resolution and thus report an upper bound for the detector resolution directly obtained from the measurements. Perpendicular to the nanowire axis an even smaller FWHM of ~150 nm was measured, which coincides well with the nanowire diameter (~150 nm). Thus, a resolution well below 100 nm should be achievable by reducing the nanowire diameter and engineering the p–n junction. In this way, direct hard X-ray detection can be established at the nanoscale, which allows for scanning high resolution direct mapping in the hard X-ray regime.

The specific shape of the XBIC signal is given by the size of the depletion zone together with the minority carrier diffusion lengths at both n- and p-doped sides^28^. Minority carrier diffusion lengths *L*_*i*_ for electrons (*L*_*D,n*_ = 68 nm) and holes (*L*_*D,p*_ = 108 nm) were extracted from the respective exponential tails (Fig. 2c; see also Supplementary Fig. 4); they are given by $L_i = \sqrt {D_i\tau _i}$, with the diffusion coefficients *D*_*i*_ and the minority carrier lifetimes *τ*_*i*_ of electrons and holes, respectively. Thus, we estimated the respective carrier lifetimes for electrons and holes to be 0.6 and 4.6 ps, respectively, using bulk values for *D*_*i*_^29^. These carrier lifetimes are much shorter compared to reported bulk values, which is due to the high recombination rate at the nanowire surface^28,30^. Although this reduces the efficiency of our hard X-ray detector, it conversely offers a very high spatial resolution. Still, a charge collection efficiency of ~0.4% can be estimated for our device (see Supplementary Fig. 4 and Supplementary Note 3), which is remarkably high considering that the interaction volume is several orders of magnitude smaller than in conventional pixel X-ray detectors^26^.

### X-ray energy dependent in-operando measurements

**Fig. 3:**
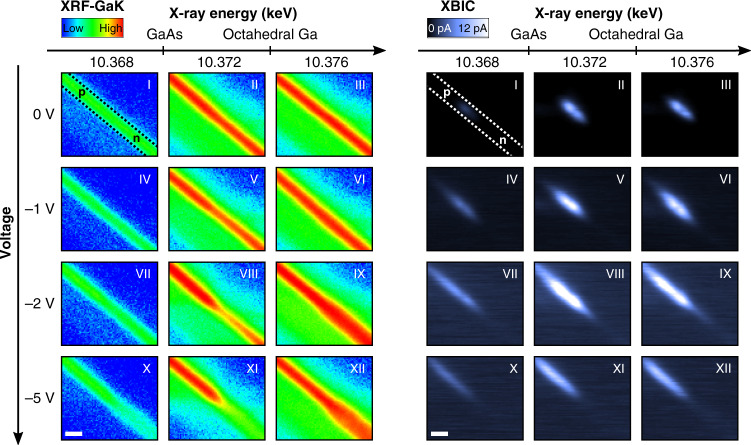
Energy and voltage dependence of nano XRF and nano XBIC. Ga K_α_ X-ray fluorescence (XRF; left) and X-ray beam induced current (XBIC; right) maps of the p–n junction taken as a function of applied voltage and excitation X-ray energy. The GaAs nanowire (as located in the Ga map) is indicated by the dashed, black and white lines, respectively. The Roman numbers indicate the measurement sequence and XRF counts and XBIC currents are scaled equally on a logarithmic scale for all voltages and energies, respectively. The spatial scale bar is 300 nm.

Further insights can be gained by scanning the incident X-ray energy across a specific absorption edge. Therefore, both nano-XRF and nano-XBIC were measured as a function of the incident X-ray energy around the Ga K-edge (Fig. 3). While XBIC maps allow to locate the p–n junction and to gain insights into the local electric fields, XRF probes the local material composition. The internal electric fields can be manipulated by applying a bias voltage to the p–n junction; furthermore, the detector charge collection efficiency can be improved by adjusting the applied voltage in reverse direction. The measurements were successively conducted for bias voltages of 0, −1, −2, and −5 V in reverse direction (indicated by negative bias voltage values on the *y*-axis). For each bias voltage, XRF and XBIC maps were recorded at different excitation X-ray energies around the Ga K-edge in steps of 1 eV, ranging from 10.367 to 10.378 keV. The XRF and XBIC maps displayed in Fig. 3 are shown for three representative incident X-ray energies, respectively, and scaled equally for all voltages and energies.

The measurements reveal several findings: the XBIC signal significantly increases for applied voltages in reverse direction due to the stronger electric field at the p–n junction, reaching its maximum at −2 V; simultaneously, however, the spatial resolution drastically decreases due to the expansion of the depletion region in the p- and the n-type segment. Conversely, increasing the voltage above −2 V, lead to a reduced XBIC signal already hinting at the degradation of the device, as shown below. Note that the nanowire device showed no degradation when it was biased in reverse direction without the X-ray beam (see Supplementary Fig. 2 and Supplementary Note 2). Therefore, both an applied bias and the incident X-ray nanobeam are necessary to induce damage.

For X-ray energies below the Ga K-edge, the secondary processes represented by XRF/XBIC signals, which result from the primary X-ray absorption mechanism, are low (first column). Increasing the incident X-ray energy across the Ga K-edge strongly increases both, the Ga K_α_ XRF as well as the XBIC signals, as visible in Fig. 3. This tendency indicates that 1 s electrons, excited by X-rays at the Ga K-edge region, contribute effectively to the electrical signal, according to the X-ray absorption process. Remarkably, above the Ga K edge-energy (middle column), the Ga K_α_ XRF signal strongly decreases with increasing voltage in the n-type region.

### XANES analysis along the p-n junction

**Fig. 4:**
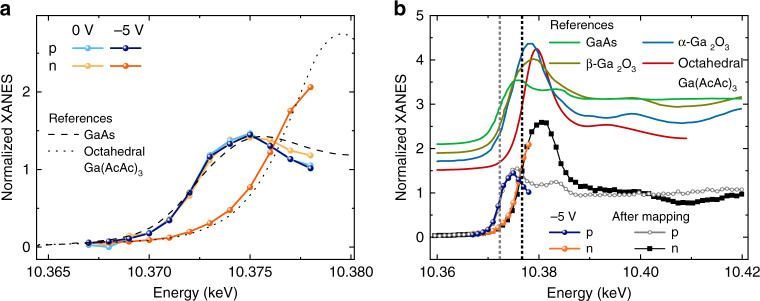
Ga K-edge XANES in the p and n-type segment. **a** Edge region of the X-ray absorption near-edge spectroscopy (XANES) spectra measured around the Ga K-edge at the p-doped (blue) and the n-doped (orange) part of the GaAs nanowire at 0 V (light colors) and −5 V (dark colors), respectively. References for GaAs and Ga in octahedral coordination (Ga(AcAc)_3_ are added as dashed and dotted lines^31,32^. A clear shift in the absorption edge can be observed in the n-doped region for the measurement at −5 V. **b** Ga K-edge XANES spectra measured after all high-resolution XBIC/XRF maps in the p and the n-type segment (black hollow circles and squares) displayed together with the edge region spectra for −5 V and reference data^31,32^. The dashed, vertical lines indicate the Ga K-edge energies from the p- and n-type segment, respectively.

To assess this signal reduction, the edge region of the XANES spectra of the GaAs nanowire for 0 V in the p- and n-type segments are plotted in Fig. 4a (light blue and light orange dots, respectively). Both the p- and the n-type GaAs nanowire segments show similar edge energies and spectral features at the Ga K-edge that are in excellent agreement with those reported for GaAs^31,32^, revealing the GaAs short-range structure at zero bias.

However, the edge region of the Ga K-edge XANES spectrum changes drastically for measurements at higher negative bias voltages. Spectra taken at −5 V from the p-and n-type segments (dark blue and dark orange in Fig. 4a, respectively) of the GaAs nanowire clearly reveal a strong shift of the X-ray absorption edge to higher energies for the n-segment, while the p-segment remains unchanged.

There are several works on Ga K-edge XANES measurements that investigate spectral energy shifts in the X-ray absorption edge^32–34^. In particular, XANES edges for Ga-based materials with different ligands and coordination states of three, four, and six were reported, and edge energies were found to increase with increasing coordination^33,35^. In GaAs, Ga has a tetragonal coordination which gives rise to the peak at at ~10.375 keV. Commonly, an increasing coordination of the Ga atoms is observed during oxidization to the stable β phase of Ga_2_O_3_, which is composed of equal fractions of tetrahedral (GaO_4_) and octahedral (GaO_6_) sites of the Ga atoms^36,37^. Thermal oxidization of GaAs has been studied in literature extensively yielding mostly β-Ga_2_O_3_, elemental As, and an As depletion^36,38,39^.

Therefore, we measured full XANES spectra without applying an external bias after the collection of high resolution XRF/XBIC maps in the p- and the n-type segments near the p–n junction (Fig. 4b); this allows to compare the observed energy shift of the Ga K-edge with literature data. Both XANES spectra are in excellent agreement with the edge region spectra taken at −5 V (blue and orange dots). Thus, the observed energy shift of the XANES edge is permanent, ruling out any transient effect associated to heat or electric fields. All XANES spectra taken in the p-type segment match the GaAs reference well. Thus, the p-type segment remained structurally unchanged throughout all experiments and still consisted of pristine GaAs. The energy shift in the n-type segment on the other hand, in comparison to β-Ga_2_O_3_ (mixed tetrahedral and octahedral), α-Ga_2_O_3_ (distorted octahedral), and Ga(AcAc)_3_ (octahedral) refs. ^31–33^, points astonishingly well to Ga(AcAc)_3_ in terms of edge energy and post-edge spectral features, representing pure octahedral coordination. The references for α- and β-Ga_2_O_3_ have XANES edges at ~2 eV lower energies (see Supplementary Fig. 6 for further reference spectra). These findings suggest a selective oxidization of the n-type segment and find Ga in a purely octahedral GaO_6_ coordination site (like in Ga(AcAc)_3_ or α-Ga_2_O_3_).

In short, following the evolution of the Ga K_α_ XRF and XBIC signals as a function of applied negative bias voltage (Figs. 3 and 4), we conclude that a step by step selective oxidization of the n-type area of the nanowire next to the p-n junction takes place. This effect is hinted by the reduction of Ga K_α_-intensity in the n-doped region with increasing negative bias voltage for the excitation X-ray energy of 10.372 keV (middle column of Fig. 3), which is between the Ga K-edges of GaAs and octahedrally coordinated Ga. Above an X-ray energy of 10.376 keV, (i.e., above the K-edge of Ga in the octahedral GaO_6_ coordination; right column in Fig. 3), the Ga K_α_-signal in the n-type segment increases again due to the selective oxidization.

**Fig. 5:**
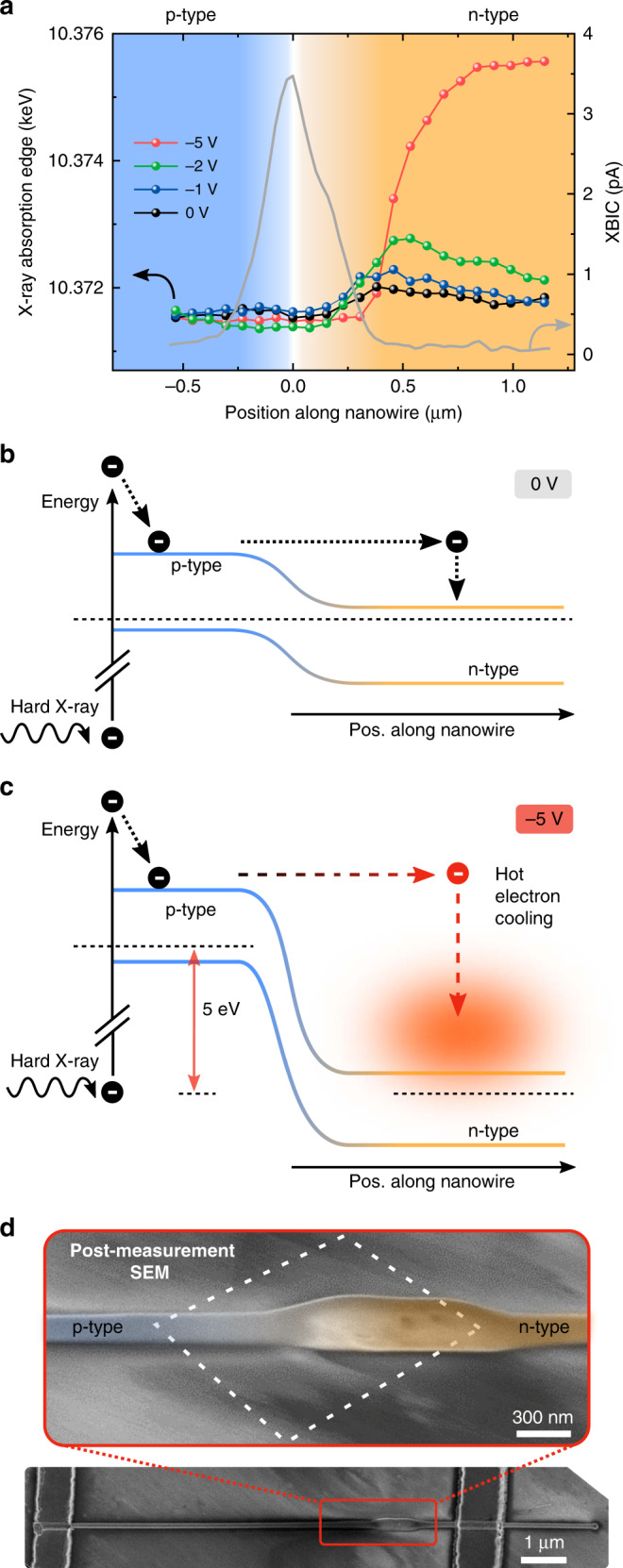
Hot electron induced selective oxidization. **a** Line scan along the nanowire of Ga K-edge energies (colored dot symbol lines on the left *y*-axis) for the different applied bias voltages. The X-ray beam induced current (XBIC) signal (grey, solid line on the right *y*-axis) for 0 V is additionally displayed to precisely locate the position of the p–n junction. **b**, **c** Schematic band diagrams along the p–n junction explaining the observed oxidization in the n-doped part due to hot electrons accelerated by the built-in voltage at the p–n junction. **d** Colorized SEM image of the device after all measurements (tilted by 50° with respect to the sample surface). The dashed line indicates the measurement region for the high-resolution XRF/XBIC maps. Below: overview SEM image of the nanowire device.

In strong contrast to these observations on the n-doped segment, no changes of the XANES energy edge were observed for the p-type side. But why is the oxidization limited to the n-type side of the p–n junction? To understand the underlying mechanisms of the oxidization, the energy of the Ga K-edge (determined from the edge region XANES spectra, see Supplementary Fig. 5 and Supplementary Note 4) along the nanowire axis is plotted as a function of the position across the p–n junction (Fig. 5a). The XBIC signal profile taken at 0 V is also displayed to exactly locate the position of the p–n junction and used as the origin of the *x*-axis (*x* = 0 μm) for all plots in Fig. 5. The Ga K-edge energy in the p-doped segment of the GaAs nanowire is constant at about 10.372 keV for all applied bias voltages. As mentioned above, the energy of the Ga K-edge also stays almost constant across the p–n junction without applied external bias voltages (0 V, black curve in Fig. 5a), revealing the pristine GaAs material. For applied bias voltages of −1 V (blue curve) and −2 V (green curve), only a slight increase of the Ga K-edge energy is visible on the n-doped side of the p–n junction. For a bias voltage of −5 V, however, the Ga K-edge energy on the n-side drastically increases up to almost 10.376 keV (red curve), as discussed above. The intermediate Ga K-edge energy values in the n-type segment most likely originate from a superposition of the GaAs core part of the nanowire with an oxidized surface layer^40^.

## Discussion

The energy band diagrams for a p–n junction without and with a bias voltage in reverse direction are depicted in Fig. 5b, c, respectively. Without an external voltage, our GaAs nanowire p–n junction has a built-in voltage of roughly 1.1 V due to band alignment with respect to the Fermi level^41^. This allows electron–hole pairs that have been created in the depletion region to be separated and measured as a current, which results in the XBIC signal displayed in Fig. 5a. Impinging X-rays excite electrons from the core levels that leave behind core-holes, which are subsequently filled from shallower levels by Auger processes or XRF. These shallower core–holes are again filled in a cascade process that, through inelastic scattering, finally leads to a larger number of electrons (holes) (~2500 per absorbed X-ray at 10.38 keV, see Supplementary Note 3) in the bottom (top) of the conduction (valence) band, as sketched in a simplified way in Fig. 5b. Electrons in the conduction band in the vicinity of the p–n junction can either recombine or “slide down” the potential to the n-type segment accelerated by the built-in potential. These hot electrons will finally transfer their excess energy to the lattice by electron–phonon scattering^42^. Yet, for low electron energies the heating of the lattice can be balanced by thermal conduction in the nanowire and to the surrounding^43^; thus, there are no significant changes of the Ga K-edge for the measurements without bias voltage (first row in Fig. 3a and the black dots in Fig. 5a). Holes will behave in a similar way, but they exhibit much lower mobilities and much faster recombination dynamic due to strongly enhanced non-radiative losses^30,44^. Thus, holes do not become as hot as electrons and no degradation in the n-type segment is observed^45^.

Charge carriers, however, are not only accelerated by the built-in voltage but additionally by an externally applied voltage. Thus, the hot electrons can in principle have energies around ~6 eV above the conduction band in the n-type region while applying a bias voltage of −5 V. Typically multiple scattering processes dominate the hot electron cooling process. However, even hot electron cooling processes beyond electron–phonon scattering were reported for high hot electron energies above 1.45 eV: inelastic impact ionization and Auger processes also become relevant factors of the hot electron energy loss in this regime^42,46^. This limits the maximum hot electron energy but greatly increases the number of electrons in the n-type segment of the p–n junction allowing for hot electron driven chemical reactions such as oxidization at the surface^46–48^. In principle, also an avalanche breakdown could follow that results in strong heating of the n-type segment of the nanowire, which possibly leads to heating and oxidization in ambient environments. In contrast, such processes are expected to be negligible on the p-type segment since, compared to electrons, holes in GaAs exhibit a mobility that is more than one order of magnitude lower and much faster recombination dynamics due to strongly enhanced non-radiative losses^30,44^. Accordingly, oxidization is only observed in the n-doped segment. GaAs has a bulk thermal conductivity of 59 Wm^−1^ K^−1^; for nanowires, an even significantly lower thermal conductivity (8–36 Wm^−1^ K^−1^) has been observed, depending on diameter and surface roughness^20,49,50^. Note that in GaAs, thermal conductivity at room temperature is not significantly influenced by doping^51,52^. Thus, the heat generated by the hot electrons cannot be efficiently transferred to the surrounding and thermal gradients can build up^43,50^. The nanowire geometry furthermore facilitates surface effects such as oxidization due to the large surface-to-volume ratio^39,53^.

The significant swelling observed at the n-side of the p–n junction, while the p-type region remained pristine (Fig. 5d) reinforces the interpretation of the selective hot electron oxidization of the GaAs nanowire. In the swollen region the nanowire diameter has roughly doubled its diameter. Energy-dispersive X-ray spectroscopy in the swollen region revealed a slight reduction of Ga and a stronger reduction of As, in good agreement with the presumed oxidization of the n-type region (see Supplementary Fig. 7 and Supplementary Note 5). We can rule out direct effects from impinging X-rays, since the measurement area of the high-resolution XRF/XBIC maps included both the p-type and the n-type segment (as indicated by the white, dashed box) and only the n-type region shows significant alteration. This is in good agreement with reports on low sample heating of contacted nanowires in an X-ray beam^43^. Furthermore, chemical effects arising from the different doping can be ruled out, since oxidization of p and n-doped GaAs was shown to be similar^54^.

Hot electrons result in the alteration of a segment with a length of ~1 µm, which is the length of the oxidized region in the n-type segment (compare Fig. 5d). Mean free paths for hot electrons in GaAs of 30–50 nm were reported in literature, however, for much lower energies compared to our case^55,56^. Increasing electron energies lead to increasing scattering rates^42,57^, such that the mean free path of highly energetic electrons should be even lower. However, the thermalization of hot electrons requires multiple inelastic scattering events; thus, electrons transfer their energy stepwise to the lattice resulting in a decreasing energy, which then leads to increasing scattering rates during the cooling process^42,55^. Therefore, multiple inelastic scattering processes during the hot electron thermalization result in the observed interaction length of ~1 µm that is several times larger than the hot electron mean free path.

In conclusion, we demonstrated the great potential of axial p–n junction nanowires as high spatial resolution hard X-ray detectors. Our device already enables a direct scanning X-ray imaging resolution of ~200 nm. Based on this approach, direct scanning X-ray imaging with tens of nanometers spatial resolution might be possible. Detailed X-ray analytical techniques enabled us to clarify hot electron effects during device operation. By combining nanoscale X-ray fluorescence and X-ray beam induced current measurements as a function of incident X-ray energy we found hot electron cooling and the subsequent oxidization to be the degradation mechanism of our biased nanowire device. Hot carrier effects are integral to a variety of devices^58^, such as Gunn diodes or IR sensors^59,60^; in transistors, however, they are also known to be a major degradation mechanism^61^. For nanoscale applications, such as nanowires, hot electron degeneration effects become even more relevant due to the limited thermal conductivity^50^ and the pronounced surface-to-volume ratio. Thus, the findings presented here provide a basis for understanding and constraining hot electron effects and oxidization in nanoscale p–n junction devices and might help establishing the heat management that is necessary for their applications as sensors and solar cells as well as their integration into nanoscale optoelectronic devices.

## Methods

All XRF, XBIC, and XANES measurements presented in this work were conducted on one single GaAs p–n junction nanowire (see the Supplementary Note 1 and Supplementary Fig. 1 for details on the growth). The X-ray measurements were conducted at the nano analysis beamline ID16B of the European Synchrotron Radiation Facility (ESRF) in Grenoble, France^21^. The flux was 2 × 10^8^ ph s^−1^ and the pulse width ~20 ps (for the bunch train in 7/8 + 1 filling mode). High resolution maps were taken by scanning the sample across the beam (scan area of 1.3 × 1.5 µm^2^, pixel size 20 nm, and integration time of 200 ms per pixel). XANES spectra were recorded with an energy resolution of 1 eV, provided by a Si (111) double crystal monochromator.

### Reporting summary

Further information on research design is available in the Nature Research Reporting Summary linked to this article.

## Supplementary information

Supplementary Information

Reporting Summary

## Data Availability

The data that support the findings of this study are available from the Corresponding Author upon reasonable request.
