# Supplementary material for: Hot electrons in a nanowire hard X-ray detector
Source: Nat Commun. 2020 Sep 18;11:4729. doi: 10.1038/s41467-020-18384-x (PMC7501287; doi:10.1038/s41467-020-18384-x)
Supplement: Supplementary file 1 — Supplementary Information [file 41467_2020_18384_MOESM1_ESM.pdf]

Supplementary Information

Hot electrons in a nanowire hard X-ray detector

Maximilian Zapf[1], Maurizio Ritzer[1], Lisa Liborius[2], Andreas Johannes[3], Martin Hafermann[1], Sven Schönherr[1], Jaime Segura-Ruiz[3], Gema Martínez-Criado[4], Werner Prost[2], and Carsten Ronning[1]**

[1] Institute of Solid State Physics, Friedrich Schiller University of Jena, Max-Wien-Platz 1, 07743 Jena, Germany

[2] Dept. Components for High Frequency Electronics and CENIDE, University of Duisburg-Essen, Lotharstr. 55, 47057 Duisburg, Germany

[3] ESRF – The European Synchrotron, 71 Avenue des Martyrs, Grenoble 30843, France

[4] Instituto de Ciencia de Materiales de Madrid, Consejo Superior de Investigaciones Científicas, Sor Juana Inés de la Cruz 3, 28049 Cantoblanco, Spain

Supplementary Figures

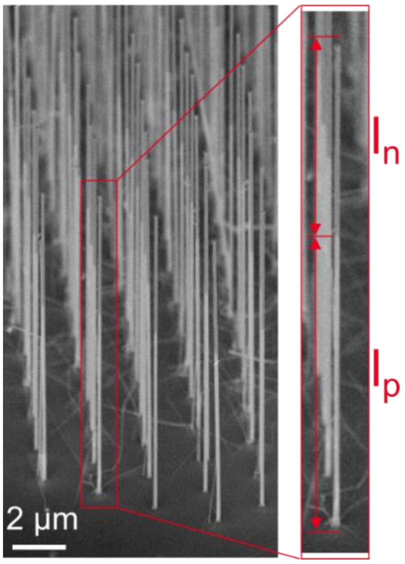

Supplementary Figure 1: Nanowire growth

SEM image of as-grown nanowires on the (111)B GaAs substrate. The n-doped part on top and the p-doped part at the bottom are marked in the inset.

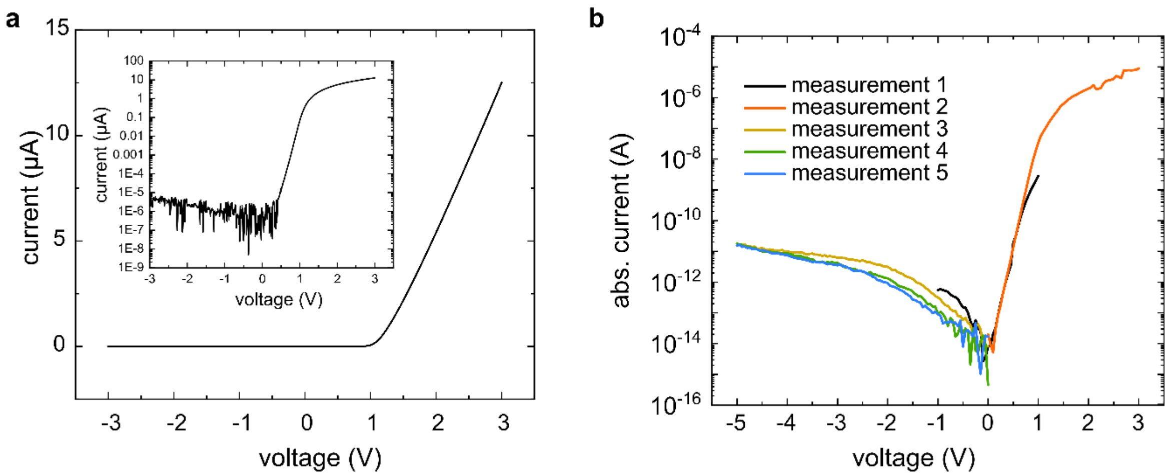

Supplementary Figure 2: Current-voltage characteristics

(a) Current-voltage characteristic of a nanowire prior to bonding. The inset shows the same data on a lin-log scale. (b) Current-voltage characteristic repeatedly swept from 0 to -5 V.

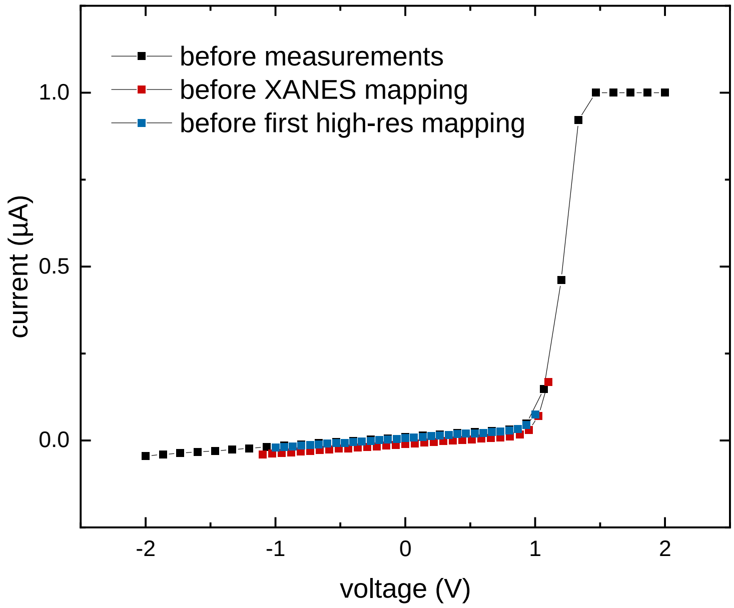

Supplementary Figure 3: Repeated current-voltage characteristics

Current-voltage characteristic of the bonded nanowire device at different stages of the experiment. The current was limited to 1 µA to protect the device.

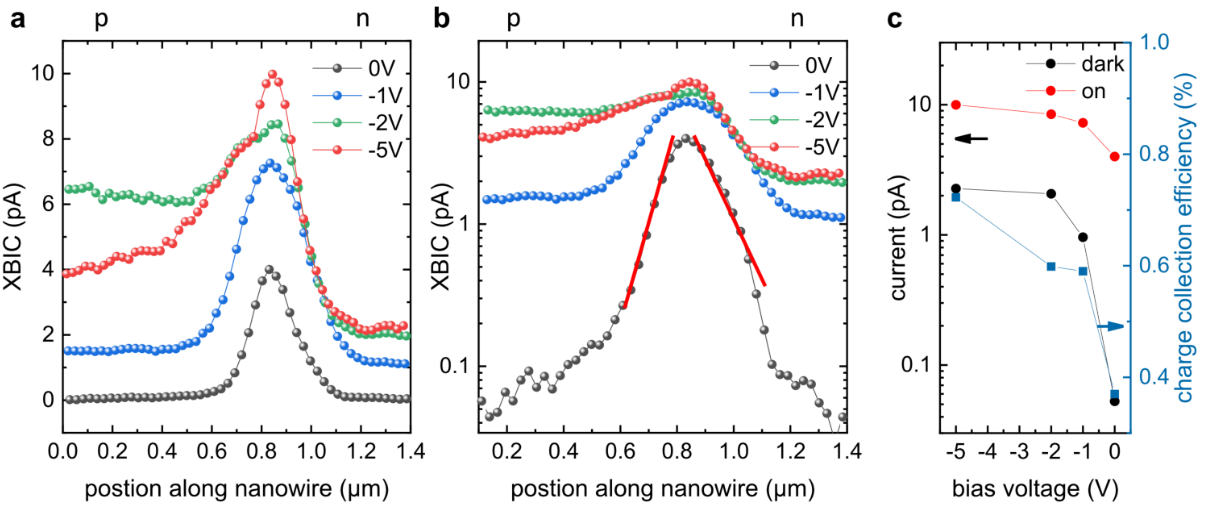

Supplementary Figure 4: Voltage-dependent X-ray beam induced current (XBIC)

XBIC line scan along the nanowire p-n junction acquired at 11.9 keV X-ray energy for different bias voltages in blocking direction on a linear (a) and log scale (b), respectively. The red lines represent the extraction of the minority carrier lengths at zero bias voltage by fitting an exponential decay to the XBIC signal. (c) XBIC maximum signal (on) and dark current (dark) as a function of the bias voltage in reverse direction. On the right, blue axis the estimated charge collection efficiency is displayed.

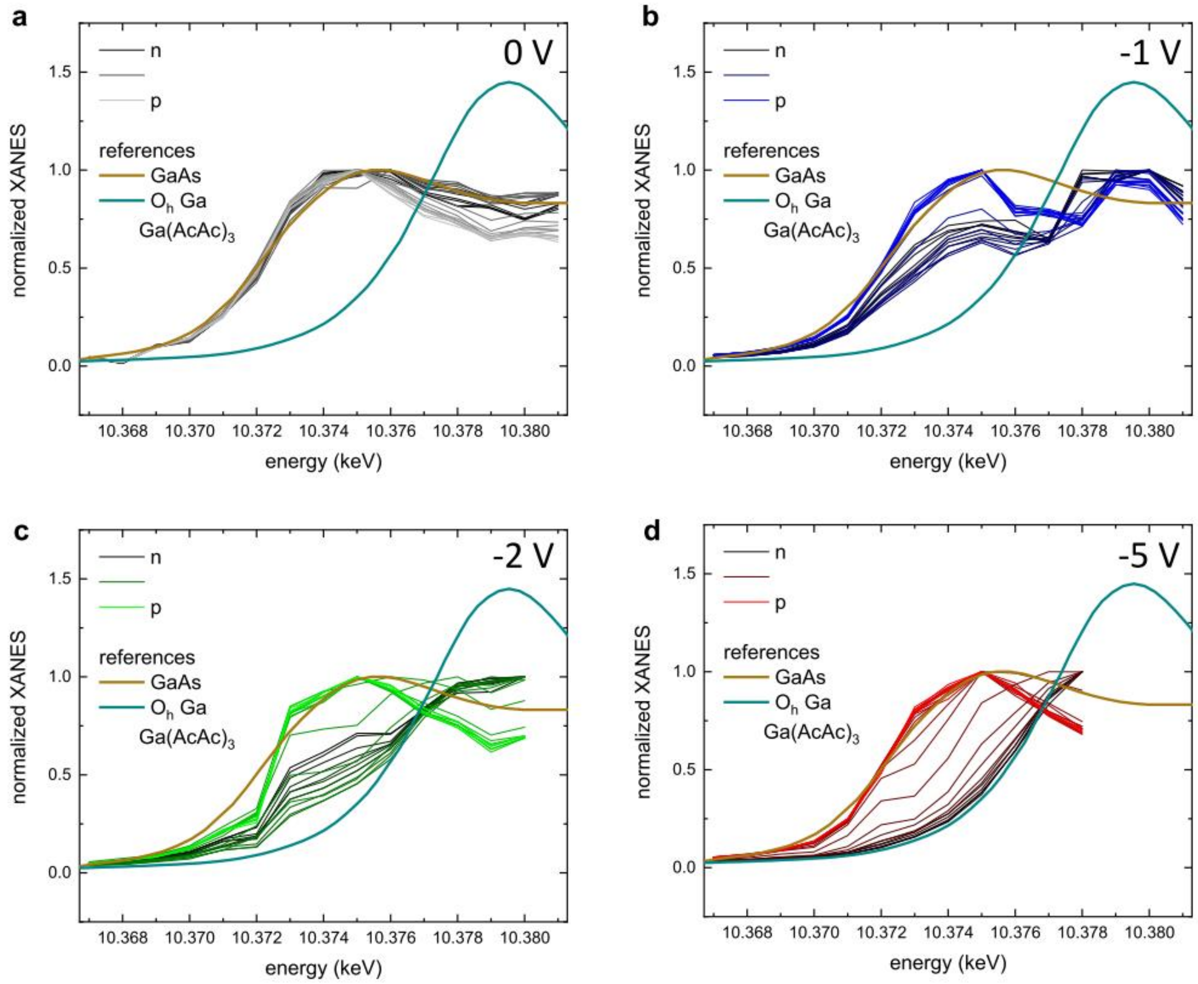

Supplementary Figure 5: Voltage-dependent X-ray absorption near-edge spectroscopy (XANES)

XANES spectra along p-n junction of the nanowire for different bias voltages. As references, GaAs and octahedtral Ga ($Ga(AcAc)_3$) are included [1,2]. For 0 V, the XANES spectra along the wire are nearly identical, proving the pristine GaAs. For increasing bias voltages, the XANES edge shifts due to oxidization. Note, that data acquisition failed for the last data points for the -5V measurement (from 10.378 keV).

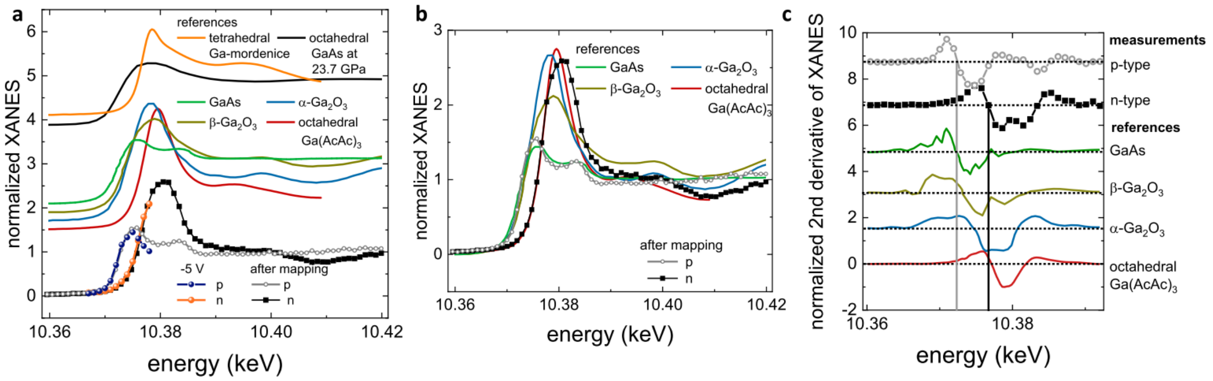

Supplementary Figure 6: X-ray absorption near-edge spectroscopy (XANES) data and references

(a) Ga K-edge XANES spectra measured after all high-resolution XBIC/XRF maps in the p and the n-type segment (black hollow circles and squares) displayed together with the edge region spectra for -5 V and reference data for GaAs, α-Ga_2O_3, β-Ga_2O_3,[1] octahedral GaAs at 23.7 GPa[3], together with references for tetrahedral Ga coordination (Ga-mordenite)[2] and octahedral Ga coordination (Ga(AcAc)$_3$).[2] (b) Selected references are shown without offset with respect to the data to allow for a clear comparison. Clearly, the hollow circles match with the GaAs reference (green) and the black data points matches best with the purely octahedral GaO_6 coordination (red). (c) Second derivatives of the respective data and references. The zero crossing (indicated by the horizontal, dotted lines), i.e., the inflection point, is taken as the edge energy. The gray, vertical lines indicate the edge energies in the p and n-type segment revealing an excellent match to the edge energies of the GaAs and octahedral Ga(AcAc)$_3$ references, respectively.

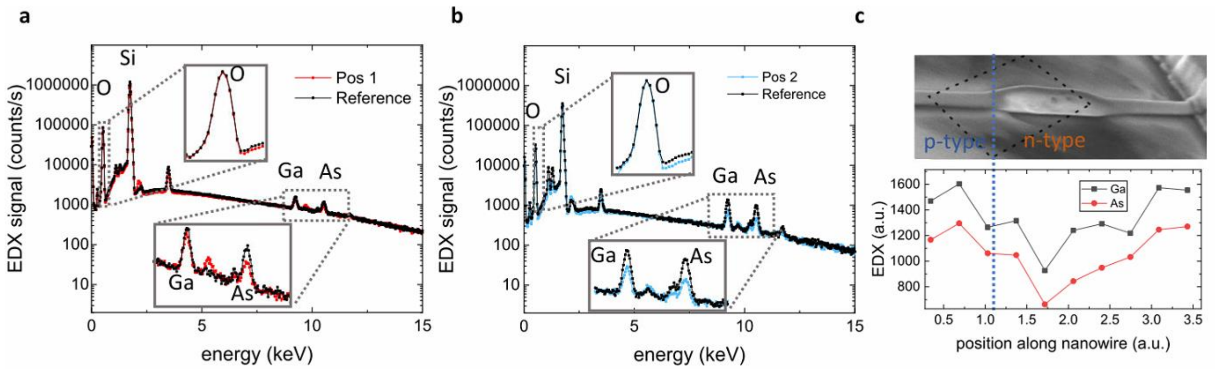

Supplementary Figure 7: Energy dispersive X-ray spectroscopy (EDX)

(a) and (b): EDX spectra taken at two different points in the swollen region. Reference spectra were recorded on the nanowire far from the swollen region. Ga and As signals are both reduced in the swollen region. (c) Lines scan of the Ga and As EDX signal along the nanowire axis. The SEM image above is aligned with the x-axis of the graph. The reduction of both signals is clearly visible in/near the swollen region. The position of the p-n junction is indicated by the blue, dashed line. The black, dashed line indicates the measurement region for the high-resolution XRF/XBIC maps.

Supplementary Notes

Supplementary Note 1: Nanowire synthesis and device fabrication

Axial GaAs nanowires were grown via vapor-liquid-solid growth mode in an AIX200 RF MOVPE reactor. Periodically structured Au-catalysts were deposited on a (111)B GaAs:Zn substrate using nanoimprint lithography (hexagonally structured mask with a hole diameter of 400 nm and 2.5 µm pitch, evaporation of 22 nm gold under 25°). Initially, the reactor was heated to T = 450 °C (50 mbar reactor pressure; 3400 mL/min total flow) under constant supply of tertiarybutylarsine (TBAs; 0.05 mmol/min) to prevent As desorption from the substrate and allow the formation of an eutectic from the Au-catalyst. Next, diethylzinc (DEZn) was supplied for 4 min to allow presaturation of the Zn-dopant in the Au-eutectic. The nanowire base is grown for 3 min using the precursors TBAs and trimethylgallium (TMGa) at a V/III ratio of 2.5 and DEZn at a II/III ratio of 0.004. After the base growth, the reactor temperature is reduced to T = 400 °C and the p-doped nanowire segment is grown for 30 min with the same precursors and precursor flows used for the base growth. The n-doped nanowire segment is grown by switching the dopant precursor to tetraethyltin (TESn) and using a IV/III ratio of 0.1. The resulting nanowires had diameters from 100 to 200 nm and lengths up to 20 µm. Prior to the wire bonding the two contacts on each nanowire were short-circuited by thin metal lines structured via electron beam lithography to prevent damage to the p-n-junction during wire bonding (electrical discharge). Afterwards, the metal lines are scraped to enable further measurements.

The doping concentrations of the p- and n-doped segment were estimated by analyzing p-GaAs and n-GaAs nanowires, grown with the same growth parameters as the p-n nanowire sections. Four individual contacts with varying distance were deposited on each nanowire and the transmission line model modified for nanowires was used. Here, the measured total resistance between contact pairs can be separated in contact and path resistances. From the latter the doping concentrations are estimated to $6 \cdot 10^{18}\,\mathrm{cm}^{-3}$ for both p- and d-doped nanowires, respectively.

After the growth, the nanowires were scraped off the growth substrate into 2-propanol and transferred dropwise onto a clean Si/SiO_2 substrate. After evaporation of the 2-propanol the nanowires are randomly distributed on the insulating substrate. Electrical contacts were defined using electron beam lithography and physical vapor deposition. On the p-type side, a Pt/Ti/Pt/Au (5/10/10/400 nm) metallization stack was deposited while for the n-side a Ge/Ni/Ge/Au (2/20/50/400 nm) stack was used. The contacts were annealed for 30 s at 280 °C in a rapid thermal annealer under nitrogen atmosphere to form ohmic contacts. For the synchrotron measurements, individual nanowire devices were macroscopically contacted by wire bonding.

Supplementary Note 2: I-V curves

Prior to bonding the nanowire devices, I-V curves of single nanowires were recorded to access the diode performance. Therefore, nanowires were transferred to an insulating Si/SiO_2 substrate (prepatterned with Ti/Au 10nm/300nm contact pads) and contacted via electron beam lithography and evaporation. I-V-measurements were performed by contacting the Ti/Au pads with gold-plated tungsten probes. Ideality factors of 2 – 2.3 and reverse saturation current densities around 3 µA/cm² were found for the measured devices. The current was normalized by the nanowire cross-sectional area. I-V measurements taken on other nanowires (more than

20) from the same growth substrate showed similar characteristics. No degradation was observed in any of the nanowires upon measuring these I-V-curves.

IV curves were repeatedly measured to monitor for device alterations. The current was limited to 1 µA and only small bias voltages of -2 to 2 or -1 to 1 V were applied. No significant changes were found during the first experiments. After the XANES high-resolution maps for a bias voltage of -5 V in reverse direction, no signal could be measured, which is in agreement with the oxidization observed in the XANES spectra.

Supplementary Note 3: XBIC measurements

The average number of created primary electron-hole pairs per absorbed X-ray is estimated by N=E/ε, with the X-ray energy E and the ionization energy ε (4.2 eV for GaAs).[4] Based on the XBIC-peak values (Supplementary Fig. 4 a and b) compared to the dark current, the charge collection efficiency CCE can be estimated by $\mathrm{CCE} = \mathrm{Q_{coll}}/\mathrm{Q_{gen}}$, where $\mathrm{Q_{coll}}$ is the collected charge as measured and $\mathrm{Q_{gen}}$ the generated charge. $\mathrm{Q_{gen}}$ can be estimated from the beam energy E and the ionization energy: $\mathrm{Q_{gen}} = \mathrm{e} \cdot \mathrm{E}/_{\varepsilon}$, with the elementary charge e.[5] At 11.9 keV X-ray energy this yields the number of generated primary electrons $N = E/_{\varepsilon} \approx 2800$ per absorbed X-ray photon (~2500 for 10.38 keV). Taking into account the absorption probability in the GaAs nanowire, the resulting charge collection efficiencies are shown in Supplementary Fig. 4 c. Effects from substrate photoelectrons were shown to be negligible in an experimental setup and device geometry comparable to our case.[6]

Supplementary Note 4: XANES measurement

Voltage dependent near edge XANES spectra are shown in Supplementary Fig. 5. All data in this plot were normalized to their maximum since no post edge was measured. References were normalized to match the findings from the full XANES spectra below.

Edge region spectra could not be normalized to the post-edge, due to the limited measurement region. We thus used the post-edge normalized full XANES spectra from the same positions to normalize the edge region spectra (see Supplementary Fig. 6). XANES edge energies can be determined as the lowest inflection point energy or from the energy of the increase to a certain fixed value. Since the latter one is more sensitive to intermediate edge values from mixed coordination states and more reliable for few measurement points at the edge region, we used it to extract the edge energies (by interpolating the measured data).

Supplementary Note 5: Energy dispersive X-ray spectroscopy (EDX) measurements

EDX measurements were performed after all synchrotron-based experiments were finished using an FEI Helios NanoLab 600i DualBeam system together with an Oxford Instruments X-Max^{N} detector. Supplementary Fig. 7 reveals a reduction of the Ga and the As signal in the swollen region, while the reduction is more pronounced in the As signal. The oxygen signal in the EDX spectrum is dominated by the signal from the insulating silica substrate underneath the nanowire. Thus, changes of the EDX oxygen signal coming from the nanowire cannot be detected by EDX. Together with the behavior of the Ga and As signals, this agrees with an oxidization of the nanowire as evidenced by XANES data.

Supplementary References

1. Martínez-Criado, G. *et al.* Crossed Ga2O3/SnO2 multiwire architecture: a local structure study with nanometer resolution. *Nano letters* **14,** 5479–5487; 10.1021/nl502156h (2014).
2. Ram Boppana, V. B., Doren, D. J. & Lobo, R. F. Analysis of Ga coordination environment in novel spinel zinc gallium oxy-nitride photocatalysts. *J. Mater. Chem.* **20,** 9787; 10.1039/C0JM01928C (2010).
3. Besson *et al.* High-pressure phase transition and phase diagram of gallium arsenide. *Physical review. B, Condensed matter* **44,** 4214–4234; 10.1103/physrevb.44.4214 (1991).
4. Alig, R. C. & Bloom, S. Electron-Hole-Pair Creation Energies in Semiconductors. *Phys. Rev. Lett.* **35,** 1522–1525; 10.1103/PhysRevLett.35.1522 (1975).
5. Lezhneva, G. M., Melkadze, R. G. & Khvedelidze, L. V. GaAs Pixel-Detector Technology for X-ray Medical Imaging: A Review. *Russ Microelectron* **34,** 229–241; 10.1007/s11180-005-0033-4 (2005).
6. Wallentin, J. *et al.* Hard X-ray detection using a single 100 nm diameter nanowire. *Nano letters* **14,** 7071–7076; 10.1021/nl5040545 (2014).
